# The Role of Arrestin Domain-Containing 3 in Regulating Endocytic Recycling and Extracellular Vesicle Sorting of Integrin β4 in Breast Cancer

**DOI:** 10.3390/cancers10120507

**Published:** 2018-12-11

**Authors:** Young Hwa Soung, Shane Ford, Cecilia Yan, Jun Chung

**Affiliations:** Department of Pathology, Stony Brook Medicine, Stony Brook, New York, NY 11794, USA; younghwa.song@stonybrookmedicine.edu (Y.H.S.); Shane.Ford@stonybrook.edu (S.F.); cecilia.yan@stonybrook.edu (C.Y.)

**Keywords:** ARRDC3, integrin β4, endocytic recycling, ubiquitination, extracellular vesicles, triple-negative breast cancer (TNBC)

## Abstract

Despite the established role of integrin β4 (ITG β4) in breast cancer progression, the importance of endocytic recycling of ITG β4 and its regulatory mechanism are poorly understood. Here, we found that a sub-population of ITG β4 is sorted into early endosomes, recycled back to the plasma membrane, and secreted in the form of extracellular vesicles (EVs) upon EGF treatment in triple negative breast cancer (TNBC) cells. A metastasis suppressor, ARRDC3 (arrestin *domain*-containing 3) prevents EGF-driven endocytic recycling of ITG β4 by inducing NEDD4-dependent ubiquitination of ITG β4 and targeting endosomal ITG β4 into lysosomes. Endocytic recycling of ITG β4 is linked to sorting of ITG β4 into EVs (ITG β4+ EVs). ITG β4+ EVs are mainly detectable from supernatants of TNBC cells and their production is inhibited by ARRDC3 expression. ARRDC3 reduces the metastatic potentials of breast cancer cell-derived EVs by reducing ITG β4 levels in EVs. Overall, current studies provide novel mechanistic insights on the regulatory mechanism of ITG β4 recycling, and its importance in invasive potentials of TNBC EVs, thus providing the basis for therapeutic targeting of the ARRDC3/ITG β4 pathway in TNBC.

## 1. Introduction

The role of integrin β4 (ITG β4) in breast cancer progression has been well established, but ITG β4 also plays a role in normal epithelia by increasing tissue integrity through formation of hemidesmosome [1,2,3]. These bi-functional roles of ITG β4 are due to the tumor micro-environment that induces phosphorylation of key Ser residues in the connecting segment of ITG β4 and subsequent disassembly of hemidesmosomes (HDs) [4,5]. Phosphorylated ITG β4 is released from HDs or endocytosed into early endosomes, and re-localized into the leading edges of migrating carcinoma cells, which allows this integrin to interact with other signaling receptors in lipid rafts [6]. ITG β4 in lipid rafts is thought to amplify signaling of nearby growth factor receptors and G protein-coupled receptors [6]. Therefore, intracellular trafficking of ITG β4 is likely to play an important role in determining signaling competency of this integrin. However, the regulatory mechanism of ITG β4 trafficking was understudied. The importance of endocytotic trafficking of integrins for their functions have been well documented by multiple reports [7,8,9]. As a classical endocytic route, integrins on the cell surface including ITG β4 are continuously internalized by clathrin-dependent or caveolar-dependent route, and rapidly enter into early endosomes (EE) [9]. Internalized integrins in EE are either directed for the recycling or degradation pathway depending on external stimuli [7,8,9]. For example, the tumor micro-environment that integrates multiple signals from matrix and growth factors is required for successful recycling of integrins, which facilitates the turnover of focal adhesions and facilitate cell motility on 2D and 3D ECM [10,11]. On the other hand, integrin could be negatively regulated by being directed to the late endosome/lysosome route for degradation [12]. The mechanism that targets endosomal ITG β4 into lysosomes is currently unknown. Endosome-driven trafficking not only contributes to receptor signaling including integrins, but also has multiple implications in regulation of metastatic potentials of extracellular vesicles such as exosomes that are of endosome origin [13,14].

Extracellular vesicles (EVs) including exosomes (30–150 nm in diameter) and micro-vesicles (150–1000 nm in diameter) mediate the interaction between cancer cells and their microenvironment, and play a critical role in development of cancers including breast cancer [13,14,15,16]. Invasive tumors take advantage of establishing a tumor environment that favors successful metastasis by secreting cancer cell derived EVs to educate neighboring as well as distantly located cells and tissues [17,18,19]. In this regard, cancer cell-derived EVs merit consideration as diagnostic markers and therapeutic targets for cancers including breast cancer [20]. A study by David Lyden’s group demonstrated that ITG β4 is found selectively in exosomes derived from metastatic breast cancer cells (mostly TNBC subtype), but not from non-invasive breast cancer cells or normal breast epithelial cells [21]. ITG β4 positive exosomes from metastatic breast cancer cells has been shown to contribute to organotropic metastasis, suggesting a role of ITG β4 in conferring metastatic potentials to cancer cell-derived exosomes [21]. However, the mechanism by which ITG β4 is sorted into EVs, and how ITG β4 contributes to metastatic potentials of cancer cell derived EVs remains to be determined.

In the current study, we tested the hypothesis that a metastastic suppressor, ARRDC3 (arrestin *domain*-containing 3) is a major regulator in determining efficiency of ITG β4 recycling and its sorting into breast cancer cell-derived EVs. ARRDC3 is one of 6 mammalian arrestins, which has been shown to possess a metastatic suppressor activity by inducing ubiquitination and degradation of phosphorylated β2-adrenergic receptor (β2 AR) and ITG β4 [22,23]. It recruits HECT-domain containing E3 ubiquitin ligases via two PPXY motifs and interacts with phosphorylated form of substrates through arrestin like domain [23,24]. While a previous report demonstrated that ARRDC3 preferentially binds to phosphorylate form of ITG β4 at S1424 [23], the identity of the E3 ligase that ubiquitinates ITG β4 is not known. Based on previous reports that ARRDC3 is a negative regulator of ITG β4 signaling [23] and its expression is epigenetically silenced in TNBC cells [25,26], we assessed the effects of modulation of ARRDC3 expression on ubiquitination of ITG β4 and its intracellular trafficking pattern in multiple subtypes of breast cancer cells. In addition, the role of ARRDC3 in sorting of ITG β4 into EVs and its implication in the metastatic potentials of breast cancer cell-derived EVs was assessed. Overall, our studies provide the mechanistic insight by which ARRDC3/ITG β4 axis regulates invasive and metastatic potentials of TNBC cells in multiple aspects.

## 2. Results

### 2.1. ARRDC3 Inhibits EGF-Driven Endocytic Recycling of ITG β4 in Triple Negative Breast Cancer (TNBC) Cells

**Figure 1:**
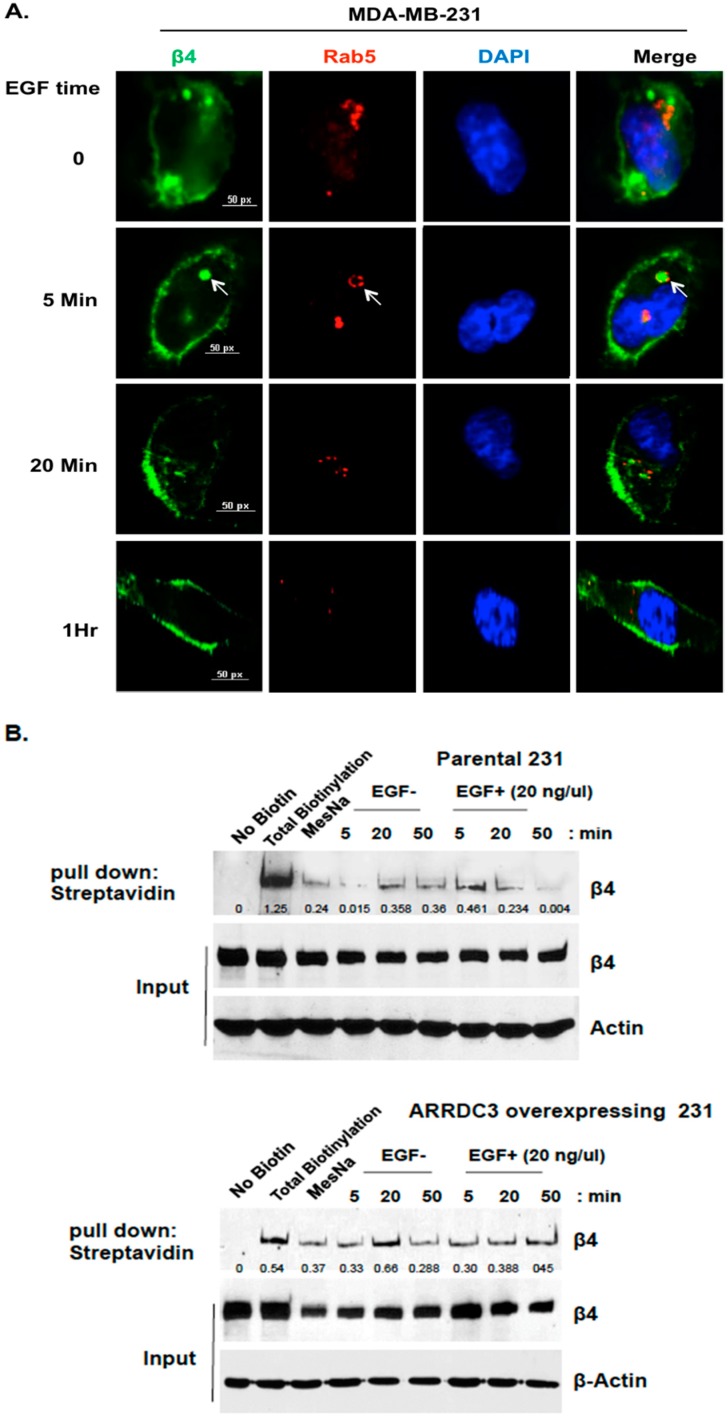

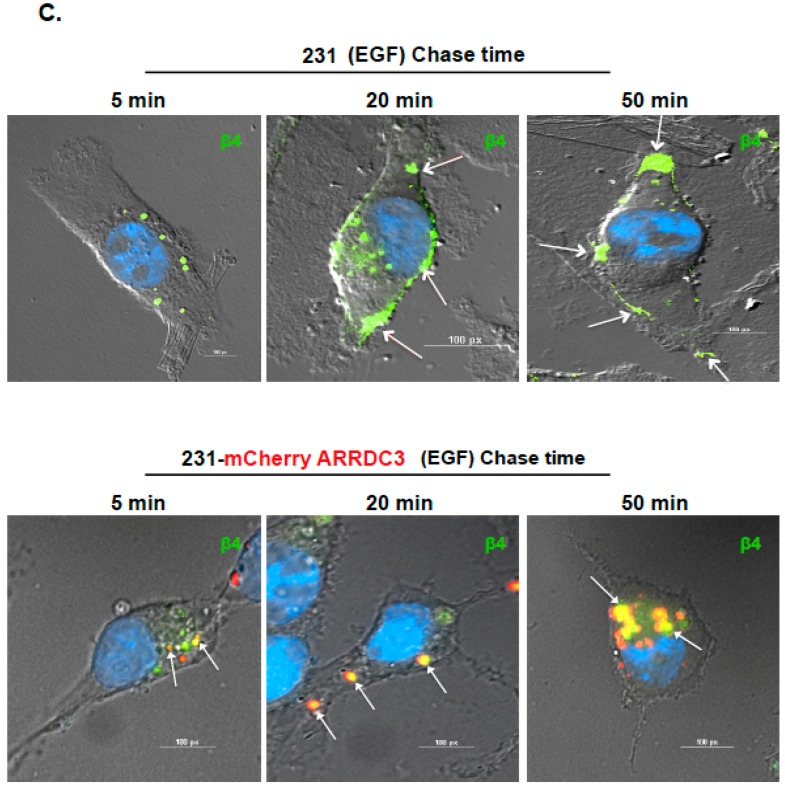
ARRDC3 inhibits EGF-driven endocytic recycling of ITG β4 in triple negative breast cancer (TNBC) cells. (**A**) MDA-MB-231 cells were serum starved for 24 h and then stimulated with EGF (20 ng/μL) for the indicated time. Cells were stained with fluorescence tagged antibodies against ITG β4 (green) or Rab5 (Red) and DAPI (for nucleus). Arrows indicate ITG β4 inside Rab5-labled early endosomes. Scale bar: 50 μm. (**B**) Biotin-based recycling assay of ITG β4 was performed in MDA-MB-231 cells with or without ARRDC3 expression as described in materials and methods section. At each chase time with EGF stimulation, cells were lysed to release biotinylated proteins. Biotinylated ITG β4 were detected by immunoprecipitation (IP). Input: whole cell lysate. Representative blots of 3 independent experiments are displayed with relative input protein (Biotinylated-β4/β4 in arbitrary unit. (**C**) MDA-MB-231 cells were transfected with or without mCherry-ARRDC3 plasmid. Immunofluorescence-based ITG β4 recycling assay were performed as described in materials and methods. Immunofluorescence signals were captured by fluorescence microscope with DIC (differential interference contrast) optic (green; ITG β4, red; ARRDC3, yellow; co-localization). Scale bar: 100 μm. Representative images were selected from three independent experiments.

A number of previous studies demonstrated the importance of endocytic recycling of integrins in their functions [7,8,9], but the mechanism that regulates integrin β4 (ITG β4) recycling and its implications in cancer biology needs to be investigated. To address this issue, we monitored the time course pattern of ITG β4 intracellular trafficking in response to growth factor stimulation in MDA-MB-231 cells (TNBC subtype cell line). As shown in Figure 1A, we observed that treatment of EGF in MDA-MB-231 cells induces the endosomal sorting of ITG β4 at 5 min as shown by the co-localization pattern between ITG β4 and Rab5 (early endosomal marker). Internalized endosomal ITG β4 is recycled back to plasma membrane as early as 20 min upon EGF stimulation (Figure 1A). EGF stimulation induced co-localization of ITG β4 with cholera toxin B, lipid raft marker and plasma membrane marker (see Supplementary Material, Figure S1). This outcome indicates that endosomal ITG β4 is going back to the plasma membrane. To obtain more definitive evidence of ITG β4 recycling, we performed cell-surface protein biotinylation based recycling assay as described previously [27,28]. Briefly, cells were labeled with HNS-SS-biotin on ice, followed by incubation with serum-free medium at 37 °C to allow internalization and removal of surface-remaining biotins by sodium 2-mercapto-ethanesulfonate (MesNa) at 4 °C. Internalized ITG β4 remain biotinylated and was detected with anti-biotin HRP linked antibody whereas recycled ITG β4 cannot be detected by anti-biotin HRP-linked antibody as surface remaining biotin was cleaved with MesNa. As shown in Figure 1B (top panel), a gradual reduction of biotinylated ITG β4 from 20–50 min time frame upon EGF treatment was consistent with its recycling pattern in Figure 1A.

On the other hand, expression of ARRDC3 in MDA-MB-231 cells did not induce a decrease in the levels of biotinylated ITG β4 even after EGF treatment, suggesting its inhibitory role in ITG β4 recycling (Figure 1B bottom panel). To confirm the role of ARRDC3 in the inhibition of ITG β4 recycling, we performed an immunofluorescence-based integrin recycling assay by using MDA-MB-231 cells transfected with mCherry tagged ARRDC3. As shown in Figure 1C, the expression of ARRDC3 prevents EGF-driven recycling of ITG β4 to the plasma membrane by sequestering ITG β4 in cytoplasm. We observed the co-localization (yellow signal) of ARRDC3 (red) and ITG β4 (green) in the cytoplasm during the time frame from 20 to 50 min after EGF stimulation, confirming cytoplasmic retention of ITG β4 by ARRDC3 (Figure 1C).

### 2.2. Inhibition of ITG β4 Recycling Is Linked to ARRDC3-Dependent Ubiquitination and Lysosomal Targeting of ITG β4

**Figure 2:**
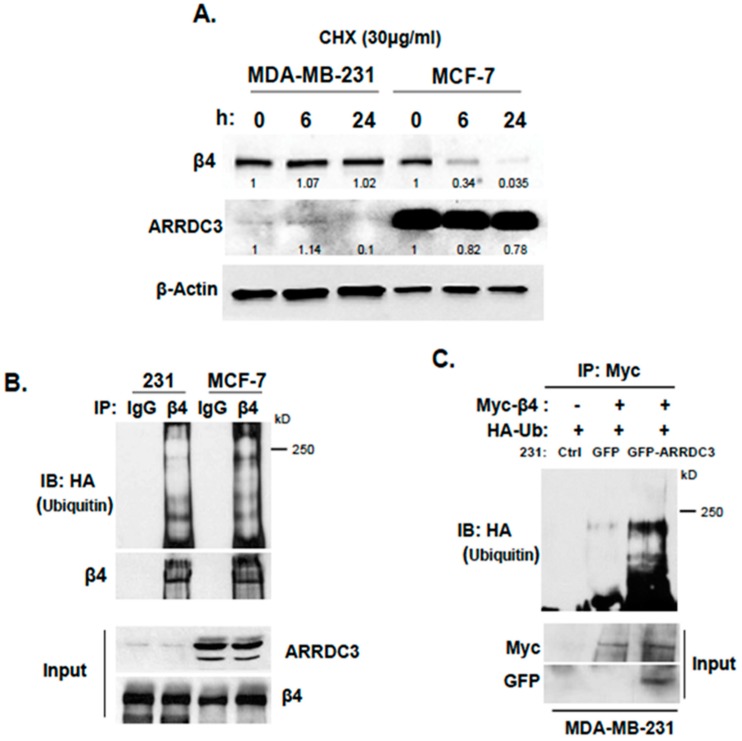

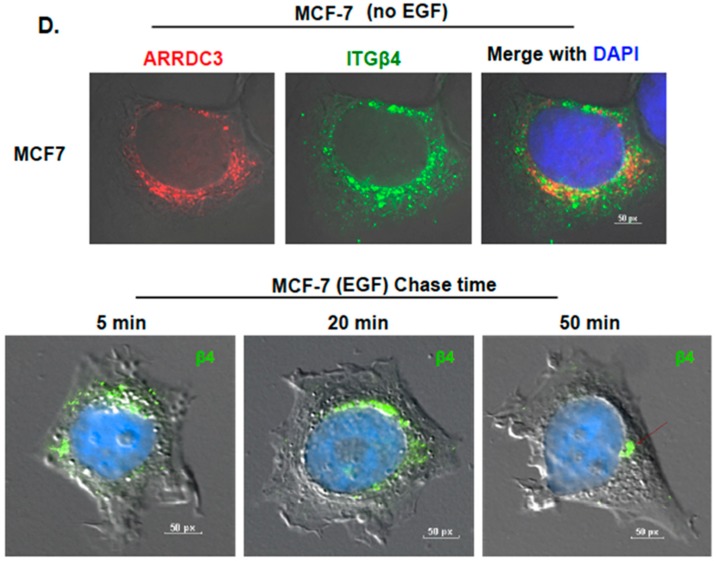
Inhibition of ITG β4 recycling is linked to ARRDC3 dependent ubiquitination. (**A**) MDA-MB-231 and MCF-7 cells were treated with 30 μg/mL of cycloheximide (CHX) for the indicated times. The levels of ITG β4 and ARRDC3 were detected by immunoblotting (IB) analysis. β-Actin was used as a loading control. (**B**) Ubiquitination of ITG β4 in MDA-MB 231 and MCF-7 cells transfected with HA-Ub was detected by immunoprecipitation (IP) with ITG β4 antibody and IB with HA antibody. (**C**) MDA-MB-231 parental and GFP or GFP-ARRDC3 expressing MDA-MB-231 cells were co-transfected with HA-Ub and either ITG β4-Myc or Myc-empty vector. IP was performed with Myc and HA trap beads. Ubiquitination was detected by IB with HA antibody. Input; whole cell lysate. All representative images were obtained from three independent experiments. (**D**) MCF-7 cells were plated on cover glasses and stained with anti-ARRDC3 (red) and anti-ITG β4 (green) antibodies, followed by mounting with DAPI (blue) (upper panel). Immunofluorescence-based ITG β4 recycling assay were performed in MCF-7 cells as described in materials and methods. Immunofluorescence signals of ITG β4 (green) were captured by a fluorescence microscope with DIC optic (lower panel). Scale bar: 50 μm. Representative images were selected from three independent experiments.

Inhibition of ITG β4 recycling by ARRDC3 suggests that ARRDC3 negatively controls intracellular trafficking of endosomal ITG β4 towards plasma membrane and instead targets endosomal ITG β4 to other intracellular compartments associated with degradation pathways such as lysosomes. To test this possibility, we measured steady-state of ITG β4 levels upon treatment of protein synthesis inhibitor, cycloheximide (CHX), in MCF7 cells that express higher levels of ARRDC3 and MDA-MB-231 cells that express very low levels of ARRDC3. The outcome showed the faster turnover of ITG β4 protein in MCF7 cells over MDA-MB-231 cells, suggesting that the stability of ITG β4 inversely correlates with the levels of ARRDC3 (Figure 2A). We then measured the levels of ITG β4 ubiquitination in these 2 cell lines by expressing HA-tagged ubiquitin (Ub), followed by immunoprecipitation of ITG β4 and immunoblotting of HA in the presence of MG132 to prevent the degradation of ITG β4. Consistent with the outcome in Figure 2A, the degree of ITG β4 ubiquitination is higher in MCF-7 cells than in MDA-MB-231 cells (Figure 2B). Over-expression of ARRDC3 in MDA-MB-231 cells increases the levels of ITG β4 ubiquitination, which further supports the hypothesis that ARRDC3 is a major determinant in ITG β4 ubiquitination (Figure 2C). We then monitored the intracellular trafficking pattern of ITG β4 in MCF-7 cells that express high endogenous levels of ARRDC3 and ITG β4 ubiquitination. Perinuclear localization of ITG β4 was observed in MCF-7 cells in the absence of external stimuli (Figure 2D). Unlike MDA-MB-231 cells, EGF treatment did not induce endosome-driven recycling of ITG β4 in MCF-7 cells (Figure 2D), confirming the inverse correlation between ARRDC3 levels and the efficiency of ITG β4 recycling. 

**Figure 3:**
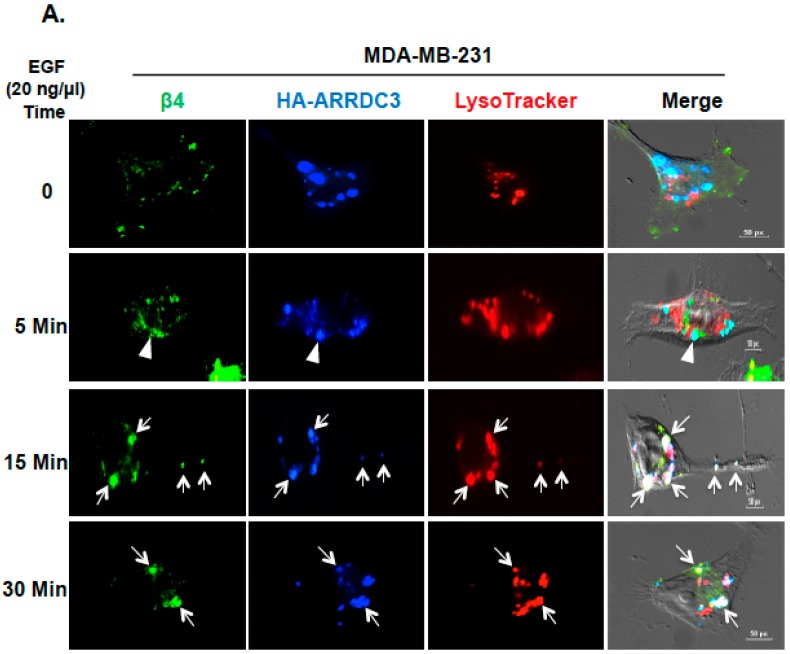

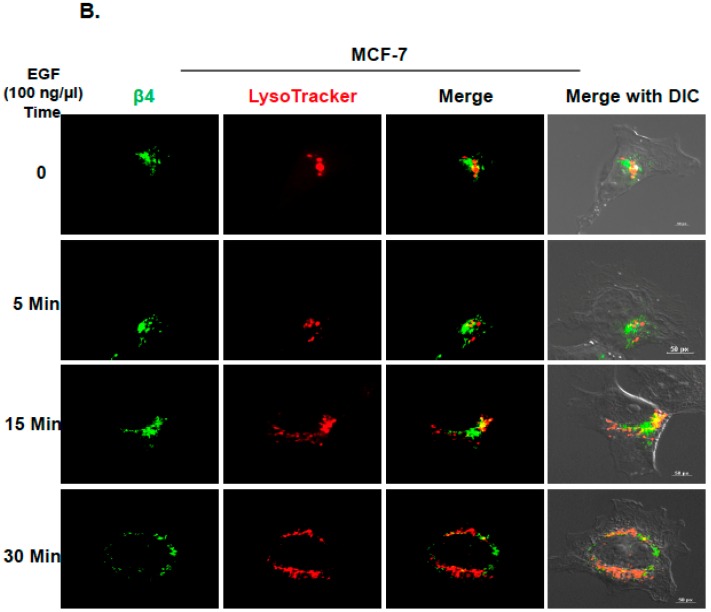
ITG β4 degradation by ARRDC3 is associated with lysosomal targeting of endosomal ITG β4. (**A**) MDA-MB-231 cells were transfected with HA-ARRDC3 plasmid. Next day, the cells were incubated with LysoTracker^®^ Red DND-99 (red) before EGF treatment (20 ng/μL) for the indicated time periods. The cells were fixed and stained with fluorescence-labeled antibodies against HA (ARRDC3; blue) and ITG β4 (green). Scale bar: 50 μm. (**B**) MCF-7 cells were incubated with LysoTracker^®^ Red DND-99 (red) before EGF treatment (100 ng/μL) for the indicated time periods. The cells were fixed and stained with fluorescence-labeled antibodies against ITG β4 (green). All images were captured by a fluorescence microscope with DIC optic. Scale bar: 50 μm. Representative images were selected from three independent experiments.

We then tested whether the cytoplasmic retention of ITG β4 by ARRDC3 is associated with lysosomal targeting of endosomal ITG β4. To address this question, we monitored the intracellular movement of ITG β4 with LysoTracker that labels late endosomes and lysosomes in MDA-MB-231 cells transfected with HA-tagged ARRDC3 (Figure 3A). Triple immuno-staining (endosomal ITG β4; green, HA (ARRDC3); blue, LysoTracker: red) showed no apparent co-localization of ITG β4 and ARRDC3 in lysosomes in the absence of EGF treatment or an earlier time point (5 min) (Figure 3A, top and second row). Co-localization of ARRDC3, ITG β4 and lysoTracker (white signal) is evident at 15–30 min after EGF treatment (Figure 3A, third-fourth rows), indicating lysosomal targeting of endosomal ITG β4 by ARRDC3. We then compared the localization pattern of ITG β4 and lysosomes in MCF-7 cells that endogenously express high levels of ARRDC3 (Figure 2B). ITG β4 remains localized in peri-nuclear regions where lysosomes are localized regardless of EGF treatment although co-localization between these two molecules is not evident in 0-5 min of EGF treatment (Figure 3B). Interestingly, co-localization between ITG β4 and lysosomes is observed at 15 min after EGF treatment. ITG β4 starts to disappear at 30 min after EGF treatment, suggesting degradation of ITG β4 in lysosomes (Figure 3B). These outcomes indicate the role of ARRDC3 in lysosomal targeting of endosomal ITG β4.

### 2.3. ARRDC3 Serves as an Adaptor Molecule for E3 Ligase NEDD4 and ITG β4 to Mediate ITG β4 Ubiquitination

**Figure 4:**
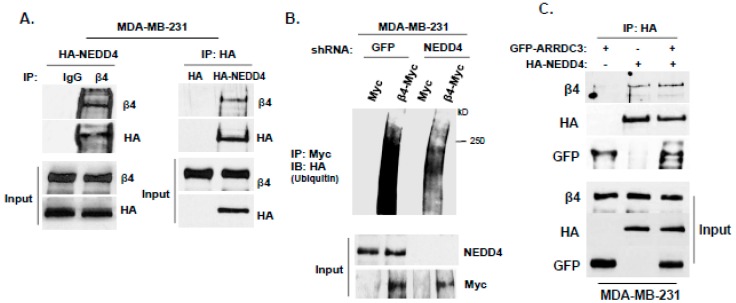

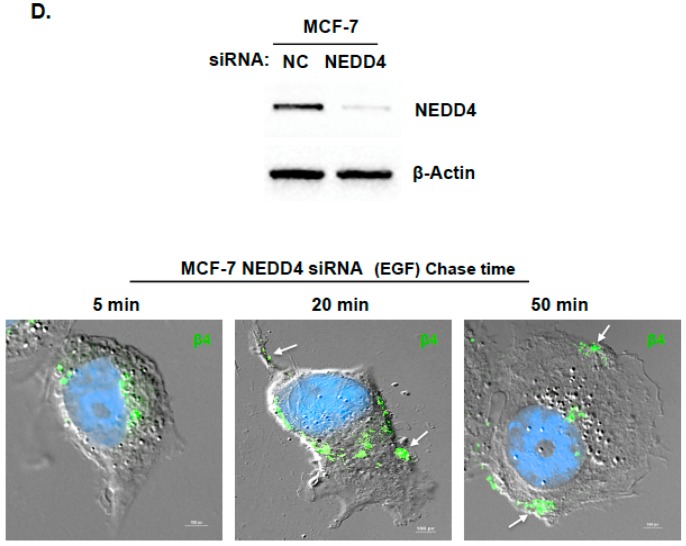
ARRDC3 mediates NEDD4 dependent ITG β4 ubiquitination by serving as an adaptor molecule between NEDD4 and ITG β4. (**A**) Endogenous ITG β4 was immunoprecipitated in MDA-MB-231 cells transfected with HA-NEDD4, followed by IB with HA (left panel). MDA-MB-231 cells were transfected with HA-empty and HA-NEDD4. IP was performed with HA trap beads, followed by IB with ITG β4 (right panel). (**B**) NEDD4 knockdown MD-MB-231 cells by shRNA were co-transfected with HA-Ub and either ITG β4-Myc or Myc-empty vector. Ubiquitination was analyzed by IP with Myc trap beads and IB with HA antibody. Input: whole cell lysate. (**C**) MDA-MB-231 cells were co-transfected with GFP or GFP-ARRDC3 and HA or HA-NEDD4. IP was performed with HA trap beads, followed by IB with ITG β4, HA and GFP antibodies. (**D**) MCF-7 cells were transfected with siRNA negative control (NC) and siRNA NEDD4. After 48 h, the knockdown was evaluated by Western blotting with NEDD4 and β-Actin antibodies (Top). Immunofluorescence-based ITG β4 recycling assay were performed in NEDD4 knockdown MCF-7 cells as described in materials and methods. Immunofluorescence signals of ITG β4 (green) were captured by a fluorescence microscope with DIC optic (bottom). Scale bar: 100 μm. All experiments were repeated at least three times.

To further define the mechanism of ITG β4 ubiquitination by ARRDC3, the identity of E3 ligase that mediates ITG β4 ubiquitination needs to be investigated. Based on the previous report that ARRDC3 serves as an adaptor between β_2_AR and E3 ligase NEDD4 to mediate β_2_AR ubiquitination [2], we assessed the role of NEDD4 in mediating ITG β4 ubiquitination. As shown in Figure 4A, HA-tagged NEDD4 co-immnuoprecipitated with ITG β4, suggesting that they interact with each other. Knockdown of NEDD4 expression by shRNA reduced the levels of ITG β4 ubiquitination (Figure 4B). The outcome indicates that NEDD4 plays a role in the ubiquitination of ITG β4. Additional co-immunoprecipitation studies involving ARRDC3, NEDD4 and ITG β4 showed that these 3 molecules form a complex to further support our original hypothesis that ARRDC3 serves as an adaptor molecule between NEDD4 and ITG β4 to mediate ITG β4 ubiquitination (Figure 4C). To further assess the role of NEDD4 in endosomal recycling of ITG β4, we knocked down expression of NEDD4 in MCF7 cells by siRNA (Figure 4D top panel). Knocking down NEDD4 expression in MCF-7 cells rescues the ability of ITG β4 to move towards the plasma membrane in response to EGF treatment (20–50 min time points) (Figure 4D bottom panel). All the evidence supports the hypothesis that ubiquitination of endosomal ITG β4 by ARRDC3/NEDD4 likely represents an important checkpoint that determines the fate of intracellular trafficking of endosomal ITG β4.

### 2.4. ARRDC3 Prevents the Sorting of ITG β4 into Extracellular Vesicles (EVs) without Affecting Overall EV Secretion from TNBC Cells

**Figure 5:**
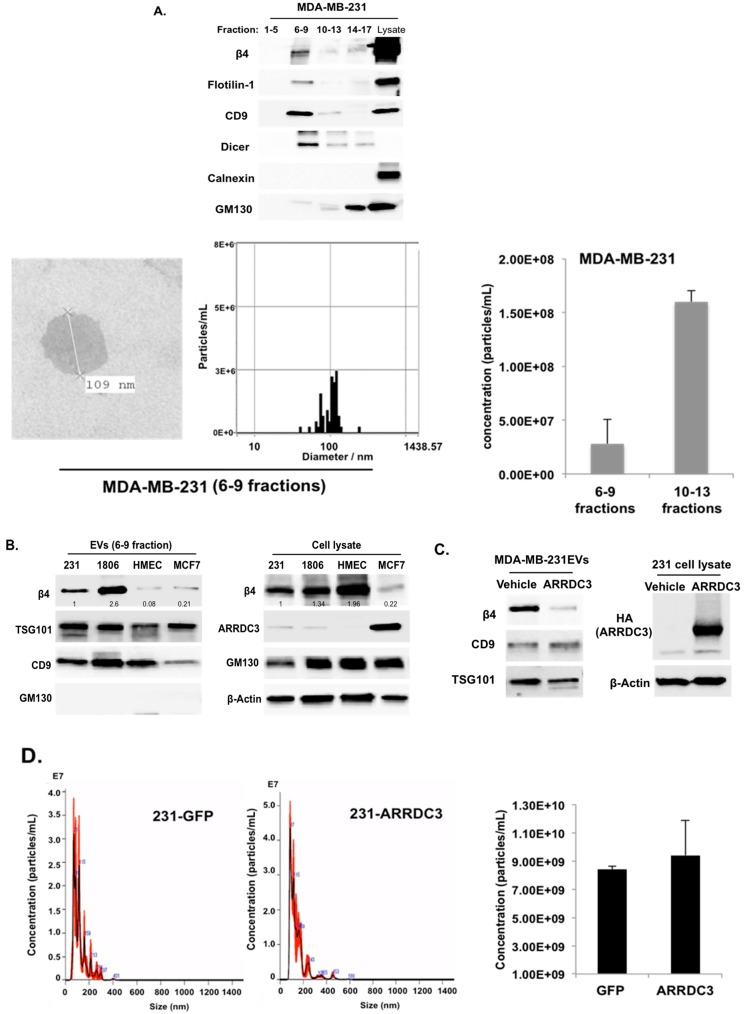
ARRDC3 prevents the sorting of ITG β4 into extracellular vesicles (EVs) without affecting overall EV secretion from TNBC cells. (**A**) EVs were isolated from MDA-MB-231 cells by using qEV column (IZON). ITG β4, exosome markers (Flotilin-1, CD9 and Dicer), non-exosome markers (Calnexin and GM-130) were analyzed by Western blot (top). Transmission electron microscope (TEM) image shows purified exosomes of MDA-MB-231 cells (bottom left). Size distribution and concentration of EVs in 6–9 fractions was measured by ZetaView (bottom right). Representative bar graph represents average concentration of selected fractions (bottom right). Data are expressed as mean ± standard deviation (SD) of three measurements. (**B**) EVs (6–9 fractions) isolated from the indicated cell lines by qEV column were concentrated by using Amicon Ultra-4. Equal amounts of proteins in EVs and total cell lysates were used for western blotting assay with the indicated antibodies. Representative blots from 3 independent experiments were displayed. Densitometric analysis was performed to measure the relative intensity of bands compared with MDA-MB-231 cells. (**C**) EVs were isolated from GFP and ARRDC3-GFP expressing MDA-MB-231 cells by using qEV column. ITG β4 and exosomal marker proteins (CD9 and TSG101) were analyzed by western blot. (**D**) Size distribution (left) and concentration (right) and of EVs isolated from MDA-MB-231 transfected with indicated plasmids were measured by NanoSight tracking analysis and analyzed by the NanoSight NTA 3.1 software. Each bar represents average values of EVs count (concentration in particles in mL) and error bars indicate SD (*n* = 9 ± SD) of the mean recorded from 3 video frames of each EVs sample for three independent experiments.

Some of the endocytosed receptors in endosomes are incorporated into intraluminal vesicles (ILV) in MVBs and are released in the form of extracellular vesicles (EVs) upon fusion of MVBs with plasma membrane [13,29]. Based on a recent report that ITG β4 is present on EVs (ITG β4+ EVs) such as exosomes secreted from metastatic breast cancer cells, and contributes to organotropic metastasis in breast cancer [21], we tested whether ARRDC3 inhibits the production of ITG β4+ EVs by preventing recycling of endosomal ITG β4. To test this hypothesis, EVs were purified using size exclusion chromatography (SEC), qEV column according to manufacturer’s protocol (IZON Science). Briefly, cell-free and EV-enriched supernatant from MDA-MB-231 cells was prepared, loaded to qEV column and eluted with phosphate-buffered saline (PBS). An equal volume of eluted fractions was used and concentrated for Western blotting analysis to monitor the presence of exosomal markers (flotillin-1, CD9, DICER), non-exosomal markers (Calnexin, GM130) and ITG β4 (Figure 5A). Both exosomal markers (flotillin-1, CD9, DICER) and ITG β4 were co-fractionated in fractions 6–9 (Figure 5A). Five independent assays consistently showed that fractions 6–9 represent exosome-enriched fractions. Nano-particle tracking analysis by Zetaview showed that average diameter of EVs fractionated from qEV columns (6–9 fractions) was 118.9 nm, and electron microscopic images confirmed the size of EVs (Figure 5A and Supplementary Figure S2).

Western blotting analysis of vesicle fractions from the qEV column (fractions of 6-9) showed that metastatic TNBC subtype cell lines (MDA-MB-231, HCC-1806) secrete higher levels of ITG β4 +EVs than non-invasive luminal subtype breast cancer cell line (MCF-7) or non-tumorigenic human mammary epithelial cell (HMEC) does (Figure 5B). HMEC has comparable levels of ITG β4 and ARRDC3 to that of TNBC cells, but its ITG β4 is not phosphorylated at Y1494 (indicator of signaling competency of ITG β4, data not shown), suggesting that only the signaling competent form of ITG β4 is incorporated into EVs. On the other hand, non-invasive MCF-7 cells express higher levels of ARRDC3 that induced degradation of ITG β4 (Figure 1), which likely explain why fewer ITG β4+ EVs were detectable (Figure 5B). Expression of ARRDC3 in MDA-MB-231 cells dramatically reduced the levels of ITG β4 in EVs (Figure 5C). Nano-particle tracking analysis of EVs isolated from MDA-MB-231 parental or GFP or ARRDC3 transfectants upon 48 h incubation of serum free culture media showed that overall EV production was not affected by ARRDC3 expression (Figure 5D), suggesting that ARRDC3 prevents the sorting of ITG β4 into EVs without affecting overall EV production. 

### 2.5. ARRDC3 Prevents EGF-Driven Fusion of CD63 Positive EVs at Plasma Membrane

**Figure 6:**
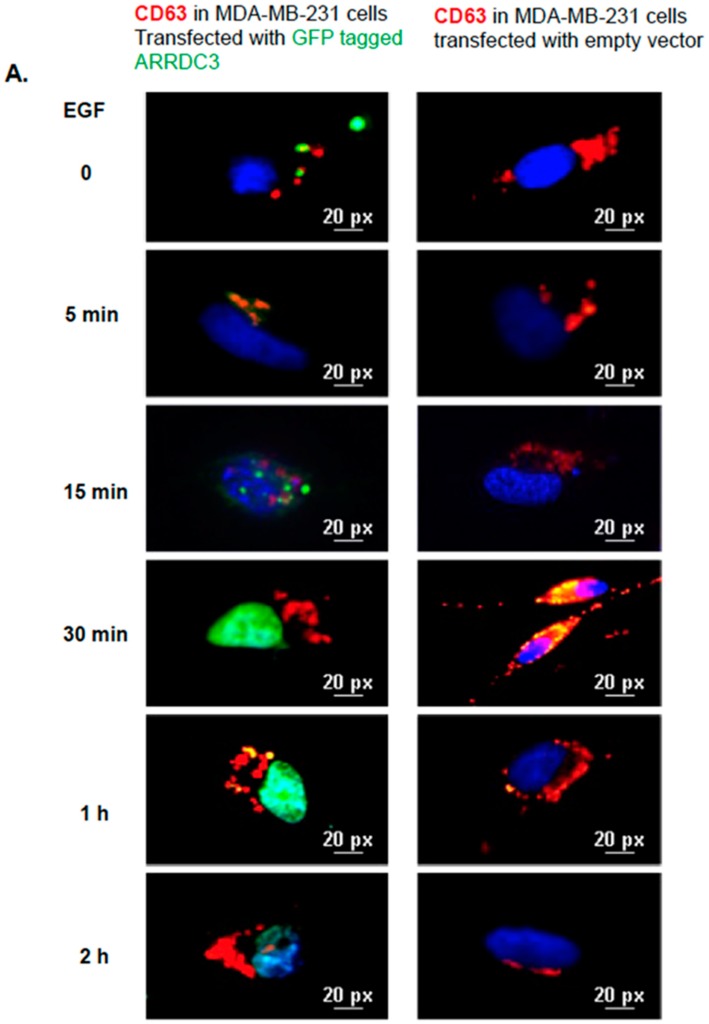

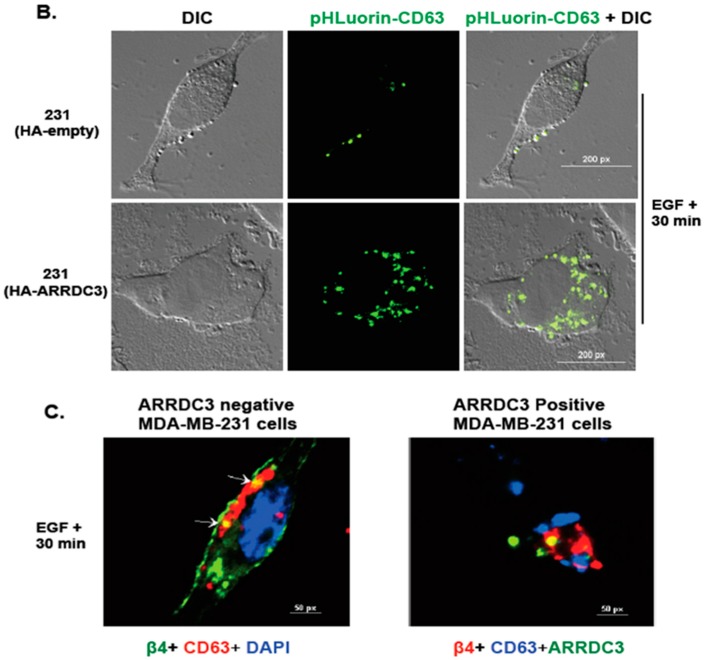
ARRDC3 prevents EGF-driven fusion of CD63 positive EVs at plasma membrane. (**A**) Immunofluorescence images show CD63 (red) and ARRDC3 (green) localization and DAPI nuclear staining (blue) in MDA-MB-231 cells with or without GFP-ARRDC3 for the time course with EGF treatment. Scale bar: 20 μm. (**B**) HA and HA-ARRDC3 expressing MDA-MB-231 cells were transfected with pHLuorin-CD63 (green) which is quenched at low pH (late endosomes) and bright at neutral pH (extracellular space or early endosome). Upon EGF treatment, CD63 location was captured by fluorescence microscope. (**C**) Immunofluorescence images show CD63 and ITG β4 localization in ARRDC3 positive and negative MDA-MB-231 cells at 30 min of EGF treatment (CD63; red, ITG β4; green, DAPI; blue: bottom left and CD63; blue, ITG β4; red, ARRDC3; green: bottom right). Scale bar: 50 μm. Representative images were selected from three independent experiments.

ARRDC3 inhibition of EGF mediated ITG β4 recycling and sorting into EVs suggests the potential role of ARRDC3 in regulating intracellular trafficking of CD63 positive EVs (mostly exosomes) upon EGF treatment. To test this hypothesis, we monitored EGF driven intracellular movements of CD63 positive EVs in MDA-MB-231 cells with or without expression of ARRDC3 (Figure 6). EGF stimulation in MDA-MB-231 cells transfected with null vector induced accumulation of CD63 signals (red) in plasma membrane including filopodia at 30 min time point, suggesting that EGF induces the fusion of CD63 positive EVs with plasma membrane as early as 30 min (Figure 6A). After 1 h, CD63 signals gradually disappeared at membrane, suggesting that they were released to extracellular space (Figure 6A). On the other hand, over expression of GFP tagged ARRDC3 (green) in MDA-MB-231 cells retains CD63 positive EVs in cytoplasm up to one hour upon EGF treatment and prevents EGF-mediated membrane fusion of CD63 positive EVs (Figure 6A). To further confirm the role of ARRDC3 in controlling cell surface localization of CD63 positive EVs by EGF, pHLuorin-CD63 (green) construct was transfected into MDA-MB-231 cells that express either null or ARRDC3. pHLuorin-GFP is pH-sensitive with a pKa of 7.1. Its fluorescence is quenched at low pH environment such as late endosomes, but bright at neutral pH, such as early endosomes or extracellular space. EGF treatment induced cell surface localization of pHLuorin-CD63 in MDA-MB-231 control transfectants at 30 min (suggesting exosome secretion), whereas ARRDC3 expression induced retention of pHLuorin-CD63 in early endosomes at the same time point upon EGF treatment (Figure 6B). Consistent with Figure 6B, both ITG β4 and CD63 were localized at the cell surface at 30 min after EGF treatment in MDA-MB-231 control transfectants, but ARRDC3 expression led to cytoplasmic retention of both ITG β4 and CD63 at the same time point (Figure 6C). Based on the result that ARRDC3 expression does not affect the overall EV production (Figure 5D), the outcome suggests that ARRDC3 is likely involved in regulation of tumor micro-environment mediated EV production (i.e., growth factors) without affecting homeostatic EV production.

### 2.6. ARRDC3 Reduces the Tumorigenic and Metastatic Potentials of TNBC Cell-Derived EVs

**Figure 7:**
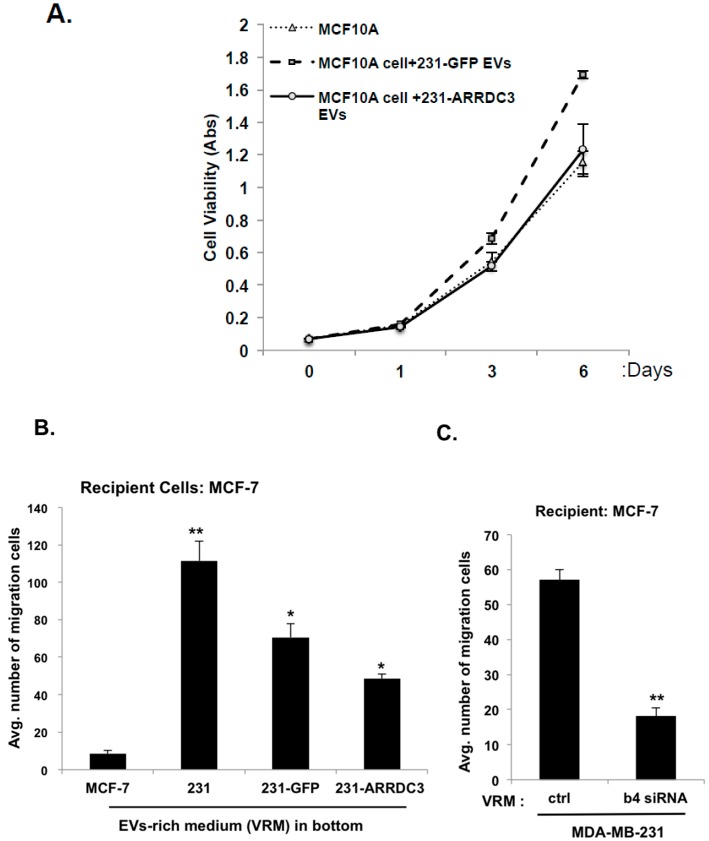
ARRDC3 reduces tumorigenic and invasive potentials of TNBC cell derived EVs. (**A**) MCF-10A cells were incubated with dil-labeled EVs derived from GFP-empty and GFP-ARRDC3 expressing MDA-MB-231 cells for the indicated times. Cell proliferation was measured by MTT assay. (**B**) MCF-7 cells were loaded into the upper chamber of a transwell. Medium containing rich EVs derived from the indicated cells (VRM; EVs rich medium) was added into the lower chamber. After 48 h incubation, the migration was quantified by counting the migrated cells to the bottom side of chamber per square milliliter using bright-field optics. (**C**) Same amounts of EVs from each MAD-MB-231 cells expressing control or ITG β4 siRNA were added to medium (VRM). The ability of MCF-7 cells to migrate under the VRM condition was measured using transwell cell motility assay. Representative results were obtained from three independent experiments. Column, mean from three independent experiments; Bars, SD. * *p* ≤ 0.005. ** *p* ≤ 0.01.

Finally, we tested whether the ARRDC3/ITG β4 axis is involved in controlling the tumorigenic and invasive potentials of EVs isolated from TNBC cells. To test this hypothesis, we measured the viability of non-tumorigenic MCF10A cells (as recipient cells) upon incubation of EVs isolated from the MCF10A cell, MDA-MB-231 cells transfected with GFP (as control) or the GFP-ARRDC3 vector (Figure 7A). While EVs isolated from GFP transfected MDA-MB-231 cells increased the viability of MCF-10A cells, EVs isolated from ARRDC3 transfected MDA-MB-231 cells did not increase the viability in comparison to EVs from MCF-10A cells (Figure 7A), suggesting that ARRDC3 expression in MDA-MB-231 cells reduced the tumorigenic potentials. Next, we measured the cell motility of non-motile MCF-7 cells (as recipient cells) by using transwell-based cell motility assays using EV-rich media in the bottom chamber (Figure 7B,C). EVs isolated from MDA-MB-231 cells increased cell motility of MCF-7 cells over 10 fold in comparison to EVs from MCF-7 cells. EVs isolated from MDA-MB-231 cells transfected ARRDC3 were less effective in stimulating cell motility of MCF-7 cells than EVs from MDA-MB-231 parental cells or control transfectants (Figure 7B). EVs from MDA-MB-231 cells whose ITG β4 expression is knocked down by siRNA was significantly less effective in stimulating the cell motility of MCF cells (Figure 7C), suggesting that ARRDC3 reduces the invasive potentials of TNBC cell-derived EVs by reducing ITG β4 levels in EVs. Overall, our studies indicate the importance of ARRDC3 in regulating metastatic potentials of breast cancer cell-derived EVs.

## 3. Discussion

Endosomal recycling of ITG β4 is a multi-step process that requires growth factor-mediated phosphorylation of ITG β4, hemidesmosome disassembly, endocytosis of ITG β4 into endosomes and endosome-mediated intracellular trafficking [5,23,30]. Here, we demonstrated that ARRDC3 is a key regulator of growth factor driven endosomal trafficking of ITG β4 by promoting NEDD4-mediated ubiquitination of ITG β4 and directing endosomal ITG β4 into lysosomes. Inhibition of ITG β4 recycling by ARRDC3 also has implications in regulating tumorigenic and invasive potential of breast cancer cell-derived EVs by reducing the levels of ITG β4+ EVs that confer invasive potentials to non-invasive recipient cells. Altogether, our results demonstrated the importance of ARRDC3/ITG β4 signaling axis in regulation of invasive potentials of TNBC in multiple aspects and provided the rationale for therapeutic targeting of ARRDC3/ITG β4 pathway.

Previous reports showed that, upon external stimuli such as growth factor receptor activation, phosphorylated and signaling competent form of ITG β4 (as evidenced by phosphorylation on key Ser and Tyr residues) moves out of hemidesmosomes and localizes into leading edges such as the lamellipodia and filopodia of carcinoma cells [4,5,30]. Therefore, it is likely that ARRDC3 preferentially inhibits endocytic recycling of signaling the competent form of ITG β4 by inducing lysosomal targeting of phosphorylated ITG β4 based on previous reports that ARRDC3 binds to the phosphorylated form of ITG β4 [23]. We expect that ARRDC3 does not affect the hemidesomosome function of ITG β4 in normal epithelial cells, as ITG β4 is not phosphorylated and, therefore, not interacting with ARRDC3. Early endosome retention and subsequent targeting of ITG β4 into lysosomes by ARRDC3 in TNBC cells strongly suggests the possibility that ARRDC3-dependent ubiquitination of ITG β4 plays an important role in determining the fates of endosomal ITG β4 trafficking. Identification of the ubiquitination sites of ITG β4 and their implications in ITG β4 signaling need to be done to further test this hypothesis in the future studies.

It is possible that ARRDC3 regulates invasive potentials of cancer EVs partially through down-regulating ITG β4+ EVs and partially through ITG β4+ EV-independent mechanisms (i.e., through regulation of exosomal miRNAs or DNAs). However, down-regulation of ITG β4+ EVs by ARRDC3 likely represent a major mechanism by which ARRDC3 reduces invasive potentials of TNBC EVs because the role of exosomal ITG β4 in metastasis was demonstrated [21], and knockdown of ITG β4 by shRNA in TNBC cells significantly reduced the ability of TNBC cell derived EVs to induce cell motility of non-motile MCF-7 cells (Figure 7C). In this regard, therapeutic approaches targeting the restoration of ARRDC3 expression merits consideration in TNBC models. Our recent studies demonstrated that small molecule inhibitors of nuclear exporter XPO1, such as selinexor, effectively inhibits the growth and invasiveness of TNBC in vitro and in vivo by up-regulating expression of ARRDC3 [26]. In this regard, small molecule inhibitors targeting ARRDC3 expression would not only inhibit TNBC functions, but also reduce the metastatic potentials of TNBC by reducing the levels of ITG β4+ EVs. As ITG β4+ EVs are mainly detectable from TNBC cell lines, potentially they could serve as liquid biopsy marker for TNBC as well as theranostic marker for drugs such as selinexor that elevates the levels of ARRDC3.

## 4. Materials and Methods

### 4.1. Cells and Reagents

MCF-7, MDA-MB-468 and MDA-MB-231 breast adenocarcinoma cells were maintained in DMEM with 1 g/L glucose, l-glutamine and sodium pyruvate formulation, supplemented with 10% FBS and 1% penicillin/streptomycin. HCC-1806, Hs578T and BT549 breast cancer cells were cultured in RPMI-1640 supplemented with 10% fetal bovine serum (FBS) and 1% penicillin/streptomycin. MCF-10A breast epithelial cells were maintained in MEGM containing 13 mg/mL BPE, 0.5 mg hydrocortisone, 10 μg/mL hEGF, 5 mg/mL insulin and 100 ng/mL Cholera toxin (Lonza, Allendale, NJ, USA). Normal HMEC (Primary Mammary Epithelial cells) cells were grown in MECM medium, supplemented with MEC growth kit (ATCC, Manassas, VA, USA). All cell lines were purchased from ATCC. They were cultured in humidified incubators at 37 °C in 5% CO_2_. 

For plasmid transfection studies, 3xHA-ARRDC3, GFP-ARRDC3 and 3xHA-NEDD4 were purchased from the GeneCopoeia, Inc. (Rockville, MD, USA). pCMV-Myc was purchased from Agilent (Santa Clara, CA, USA). Integrin β4 cDNA was cloned into pEGFP-N2 and pCMV-Myc respectively. pHLuorin-CD63 was a gift from Dr. Maarten Bebelman (Vrije University, Amsterdam, Netherlands). The transfection of all plasmids was carried out using Lipofectamin LTX-Plus (Invitrogen, Grand Island, NY, USA) or Lipofectamin 3000 (Invitrogen).

Integrin β4 (sc-9090), HSP70 (sc-24), Dicer (sc-30226) and β-actin (sc-1615) antibodies were purchased from Santa Cruz Biotechnology (Santa Cruz, CA, USA). GFP monoclonal antibody was obtained from Clontech (Mountain View, CA, USA). ARRDC3 (ab64817), Rab5 (ab109534), TSG-101 (ab30871), CD63 (ab68418) and CD9 (ab3223) antibodies were obtained from Abcam (Cambridge, MA, USA). CD104 (BD555719), Flotilin-1 (BD610820) and GM130 (BD610822) were purchased from BD Biosciences (San Jose, CA, USA). NEDD4 (#2740), Calnexin (#2679), Myc-Tag (#2272), HA-Tag (#2367 or #3724) antibodies were purchased from Cell Signaling.

MG 132 (proteasome inhibitors) and Cyclohexamide (CHX; protein synthesis inhibitor) were purchased from Sigma (St. Louis, MO, USA). EGF was obtained from Sigma-Aldrich and Lysotracker red DND-99 was purchased from Invitrogen.

siRNAs were purchased from Ambion (Thermo Fisher Scientific, Waltham, MA, USA) and used to target human integrin β4 (sense GCGACUACACUAUUGGAUUtt and antisense AAUCCAAUAGUGUAGUCG Ctg) and human NEDD4 (assay ID; s9417) Cells were plated on dish for 24 h before siRNA transfection. Transfection was performed using RNAiMAX reagent (Invitrogen) according to the manufacturer’s instructions. To enhance knockdown, cells were typically grown for 3 days. To generate stable NEDD4 knockdown cell lines, MDA-MB-231 cells were infected with lentiviruses expressing shRNA targeted against NEDD4 or GFP as control (Sigma). The infected cells were then selected by puromycin (20 μg/mL).

### 4.2. Western Blot Analysis

Cells were lysed in cold radioimmunoprecipitation assay–ethylenediaminetetraacetic acid (RIPA–EDTA) buffer (50 mM Tris, pH 7.4; 150 mM NaCl; 1% NP-40; 0.5% sodium deoxycholate; 0.1% SDS; and 5 mM EDTA) containing 1 mM phenylmethylsulfonyl fluoride, 1 mM Na_3_VO_4_, and protease inhibitor (Thermo Scientific Pierce, Rockford, IL, USA). The protein concentrations were determined using the BCA protein assay kit (Thermo Scientific Pierce). The samples were separated on 4% to 20% gradient SDS PAGE and transferred to polyvinylidene difluoride (PVDF) membranes by using the Trans-Blot Turbo transfer system (Bio-Rad, Hercules, CA, USA). The blots were incubated with primary antibodies in TBS-T or TBS-T with 5% *w*/*v* nonfat dry milk, then with appropriate secondary antibodies conjugated to IgG-horseradish peroxidase. Proteins were detected using the Clarity Western ECL blotting substrate (Bio-Rad). All bands were imaged with ChemiDoc Touch Imaging System (Bio-Rad).

### 4.3. Ubiquitination and Co-Immunoprecipitation Assay

Cells were seeded in 100 mm dishes and were transiently transfected with indicated plasmids using Lipofectamin 3000 or LTX-plus lipofectamin (Invitrogen). For β4 uiquitination, cells expressing the vehicle or β4 with HA-ubiquitin were treated with 10 M MG132 for 6 h. The cells were lysed in CellLytic M cell lysis buffer (Sigma). Protein lysates were incubated with antibodies and protein G agarose (Sigma) overnight at 4 °C. HA- or Myc-tagged proteins were immunnoprecipitated with anti-HA or anti-Myc-coupled beads using anti-HA IP kit and anti-c-Myc IP kit (Sigma) according to the manufacturer’s protocol. Beads were washed five times with IP buffer and boiled in Laemmli sample buffer (Bio-Rad). The samples were subjected to sodium dodecyl sulphate-polyacrylamide gel electrophoresis (SDS-PAGE) and Western blot analysis.

### 4.4. Assays That Monitor Intracellular Trafficking of ITG β4

#### 4.4.1. Biotin-IP Based Integrin Recycling Assay

Cells were grown to 80–90% confluence in a 6-well plate were placed on ice and washed twice with ice cold PBS. Cell surface proteins were labeled with 0.5 mg/mL NHS-SS-biotin (Thermo Fisher Scientific) in PBS at 4 °C for 40 min. Unbound biotin was gently washed away in cold PBS containing 100 mM glycine. Labeled cells were transferred to pre-warmed serum-free medium and incubated at 37 °C for 30 min to allow internalization. After two washes with cold PBS, surface-remaining biotin was removed by cleavage with 100 mM MesNa (sodium 2-mercapto-ethanesulfonate) at 4 °C for 25 min. Cells were washed with ice-cold PBS and excess biotin was quenched with 5 mg/mL iodoacetamide for 10 min on ice. To allow integrin recycling, MesNa treated cells were incubated at 37 °C for different times (5, 20, 50 min). The cells were returned to ice, and biotin at the cell surface was removed by MesNa treatment. After washing with cold-PBS, cells were lysed in RIPA buffer and then biotinylated proteins were isolated from cells extract by immunoprecipitation on streptavitin-agarose beads (GE Healthcare, Chicago, IL, USA). Washed beads were eluted with SDS sample buffer and eluted proteins were subjected to immunoblotting analysis.

#### 4.4.2. Immunofluorescence-Based Integrin Recycling Assay

Cells were grown on coverslips and serum starved at 37 °C overnight. The cells were transferred to ice to cool down. Surface integrin β4 were labeled with anti-Alexa 488-CD104 antibody (Invitrogen, #MA5-23641) in serum-free medium containing 0.01% bovine serum albumin (BSA) for 1h at 4 °C. Labeled cells were washed two times with cold serum-free medium containing 0.01% BSA and subsequently transferred to prewarmed free-serum medium. Integrin internalization was allowed at 37 °C for 2 h. For recycling, internalized integrin was stimulated with EGF for different times at 37 °C. The cells were washed twice with PBS, fixed in paraformaldehyde (PFA) and mounted with Fluoromount-G (Southern Biothech, Birmingham, AL, USA) or Vectashield DAPI (Vector lab, Burlingame, CA, USA) for. Integrin trafficking was monitored by immunofluorescence microscope. Protein localization was captured at 60× oil magnification and DIC was captured at CFI Plan Apo Lambda 60× Oil magnification using a Nikcon Eclipse Ts2R microscope with Nikon DSQi2 Digital Camera. All images were analyzed using NIS-Elements software (NIS-Elements advanced research 4.5 version, Nikon, Tokyo, Japan) and processed using Adobe Photoshop software (Adobe Photoshop CC 2015).

### 4.5. EVs Purification

For size exclusion chromatography (SEC) methods, cells were grown to 60–70% confluences, washed with PBS and incubated with a minimal volume of serum-free medium required to cover the cells. After 48 h, cell supernatant was centrifuged (350 *g* for 10 min and 2000 *g* for 30 min) to remove cells and debris, followed by filtering with 0.22 μm filter (Millipore, Burlington, MA, USA). The cell-free supernatant was concentrated to 100–200 μL with using Amicon Ultra-4 10 kDa filter (Millipore). For EV (mostly exosome) isolation, 100 μL of concentrated cell supernatants were subjected to SEC by qEV column (IZON Science Ltd., Cambridge, MA, USA). Briefly, the column was rinsed with 10 mL of filtered PBS before use. The samples were layered onto the top of qEV column followed by elution with PBS. The first 5 fractions (total 1 mL) were discarded (this void volume does not contain EVs). Subsequently, each 200 μL fraction (fractions from 6 to 17) was collected. The isolated EV samples were frozen at −80 °C. 7–9 fractions containing high EVs were pooled and filtered for subsequent assay. The size, concentration and Zeta-potential of EVs were measured using ZetaView (Particle Metrix, Germany) and NanoSight (Malvern Panalytical, UK) nanoparticle tracking analysis.

### 4.6. Transmission Electron Microscopy (TEM)

EV quantity was measured via ZetaView (Particle metrix GmbH, Microtrac, Meerbusch, Germany) and each EV sample was fixed in 2% paraformaldehyde. This sample was adsorbed for 1 min to a carbon-coated grid. Excess liquid was removed with filter paper and the sample was stained with 0.75% uranly formate for 30 s. The excess uranly formate was removed with filter paper and the grid was observed on a FEI BioTwinG2 transmission electron microscope at 120 kV. All images were captured by AMT XR-60 CCD digital camera system (AMT, Woburn, MA, USA). 

### 4.7. Proliferation and Transwell Migration Assay

Recipient cells plated in 96 well plates were incubated with purified EVs for indicated time. Proliferation assay was measured using the CCK-8 kit (Dojundo Molecular Technologies) according to the manufacturer’s instructions. Absorption at 450nm was determined using an iMark Microplate Reader (Bio-Rad). For the cell migration assay, recipient cells were plated into insert of transwell cell culture chamber with 8 μm pore size (Costar-BD Falcon) according to the standard procedure. To generate EV-rich medium, 50 μg of EVs isolated through size exclusion chromatography were diluted in serum-free medium. EV-rich medium was added in the lower chamber as a chemoattractant and cells were incubated at 37 °C for 24 h. The migrated cells on the lower surface of the membrane were fixed and stained with 0.2% crystal violet and counted. Assays were performed in triplicate and repeated three times.

### 4.8. Statistical Analysis

Analytical studies were typically performed several times in independent experiments. Data were statistically analyzed using GraphPad Prism and ImageJ software (https://imagej.nih.gov/ij/). All quantitative data are presented as mean ± standard deviation (SD). Student’s *t* tests were used for comparisons of means of quantitative data between groups. Significance was set to *, *p* ≤ 0.005; **, *p* ≤ 0.01.

## 5. Conclusions

In summary, we have shown that ARRDC3 is a key regulator of endosomal recycling of ITG β4 and production of ITG β4+ EVs. These data identify novel mechanisms by which the fate of interluminal vesicles in late endosomes is determined and the invasive potential of cancer cell-derived EVs is regulated. Our studies provide the basis for a theranostic strategy targeting the ARRDC3/ITG β4 pathway in TNBC.

## Supplementary Materials

The following are available online at http://www.mdpi.com/2072-6694/10/12/507/s1, Figure S1: MDA-MB-231 cells were stimulated with or without EGF (20 ng/mL) for 20 min, then surface were labeled with CT-B (cholear toxin subunit B used as a marker for lipid rafts, which are membrane microdomains)-Alexa 594 (red). Figure S2: The particle size (nm) was measured using the ZetaView for NTA. Representative bar graph shows average size of selected fractions.

## Abbreviations

ARRDC3	Arrestin domain-containing 3
ITG4	Integrin 4
TNBC	Triple negative breast cancer
Evs	Extracellular vesicles
NEDD4	Neural precursor cell expressed, developmentally down-regulated 4
E3	Ubiquitin protein ligase 3
IP	Immunoprecipitation
