# Supplementary material for: The Role of Arrestin Domain-Containing 3 in Regulating Endocytic Recycling and Extracellular Vesicle Sorting of Integrin β4 in Breast Cancer
Source: Cancers (Basel). 2018 Dec 11;10(12):507. doi: 10.3390/cancers10120507 (PMC6315883; doi:10.3390/cancers10120507)
Supplement: Supplementary file 1 [file cancers-10-00507-s001.pdf]

Supplementary Materials: The Role of Arrestin Domain-Containing 3 in Regulating Endocytic Recycling and Extracellular Vesicle Sorting of Integrin β4 in Breast Cancer

Young Hwa Soung, Shane Ford, Cecilia Yan and Jun Chung

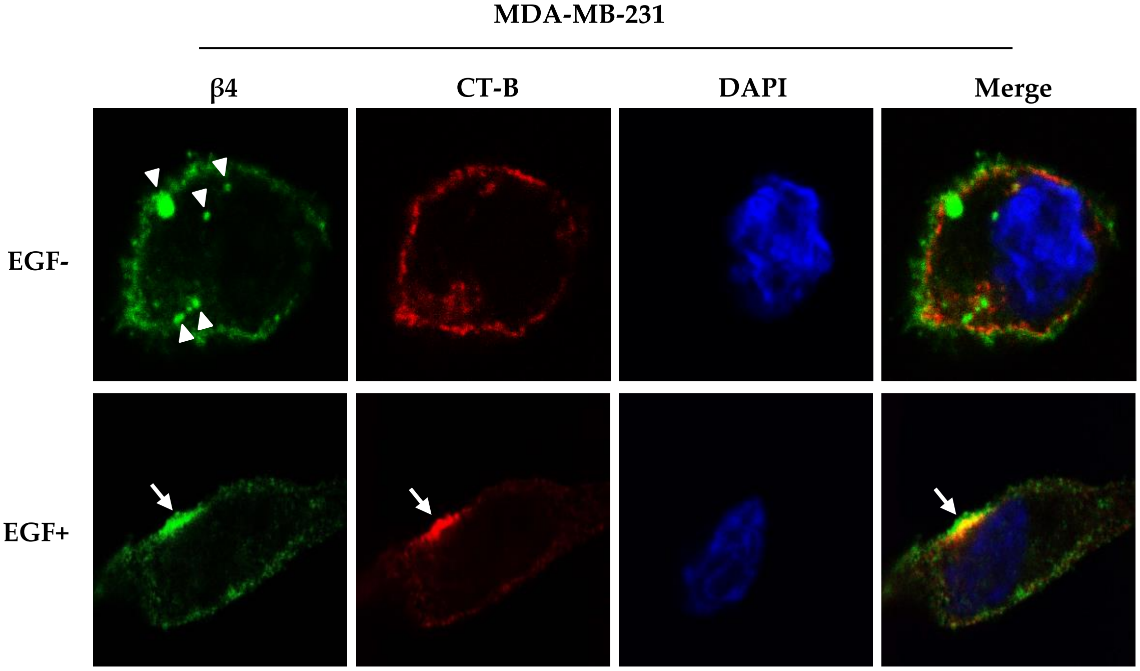

Figure S1. MDA-MB-231 cells were stimulated with or without EGF (20 ng/mL) for 20 min, then surface were labeled with CT-B (cholear toxin subunit B used as a marker for lipid rafts, which are membrane microdomains)-Alexa 594 (red). Cells were fixed and stained with ITG β4 (green). All images were captured by fluorescence microscope. Arrows indicates that ITG β4 is co-localized with CT-B to membrane. Arrowheads indicate ITG β4 located in hemidesmosomes (HD). Representative images were selected from three independent experiments.

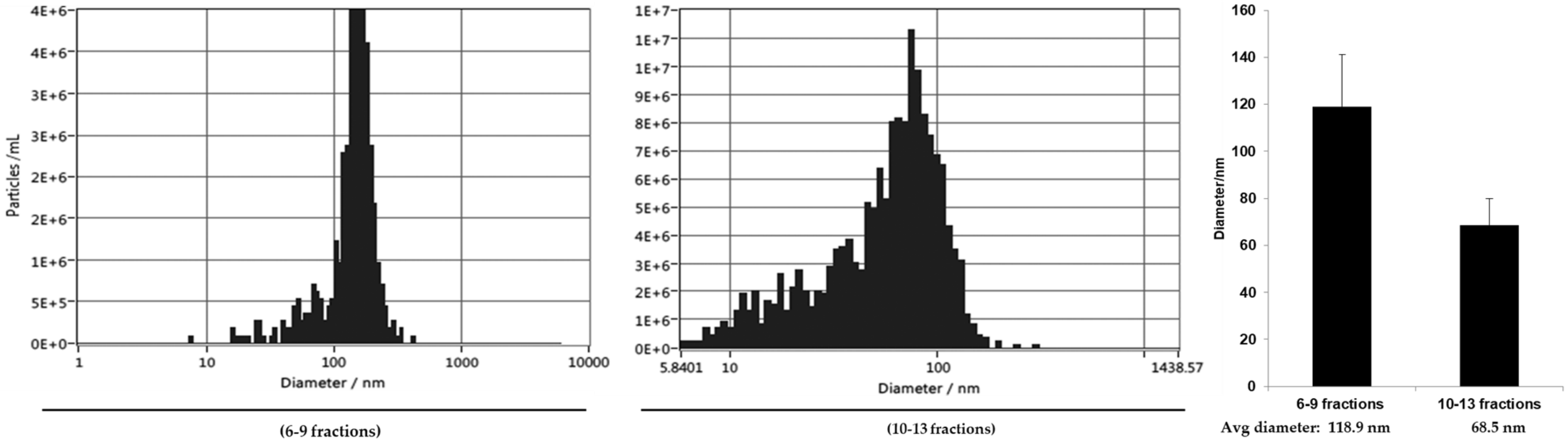

Figure S2. The particle size (nm) was measured using the ZetaView for NTA. Representative bar graph shows average size of selected fractions. Data are expressed as mean ± SD of three measurements.

© 2018 by the authors. Licensee MDPI, Basel, Switzerland. This article is an open access article distributed under the terms and conditions of the Creative Commons Attribution (CC BY) license (http://creativecommons.org/licenses/by/4.0/).
